# Identification of potential therapeutic targets in a model of neuropathic pain

**DOI:** 10.3389/fgene.2014.00131

**Published:** 2014-05-23

**Authors:** Hemalatha B. Raju, Zoe Englander, Enrico Capobianco, Nicholas F. Tsinoremas, Jessica K. Lerch

**Affiliations:** ^1^Center for Computational Science, Department of Medicine, University of Miami Miller School of MedicineMiami, FL, USA; ^2^Human Genetics and Genomics Graduate Program, University of Miami Miller School of MedicineMiami, FL, USA; ^3^Department of Biomedical Engineering, Duke UniversityDurham, NC, USA; ^4^Laboratory of Integrative Systems Medicine, National Research Council (CNR)Pisa, Italy; ^5^Department of Neuroscience, Center for Brain and Spinal Cord Repair, The Ohio State UniversityColumbus, OH, USA

**Keywords:** gene expression, neuropathic pain, spinal cord injury, dorsal root ganglia, sciatic nerve, RNA

## Abstract

Neuropathic pain (NP) is caused by damage to the nervous system, resulting in dysfunction and aberrant pain. The cellular functions (e.g., peripheral neuron spinal cord innervation, neuronal excitability) associated with NP often develop over time and are likely associated with gene expression changes. Gene expression studies on the cells involved in NP (e.g., sensory dorsal root ganglion neurons) are publically available; the mining of these studies may enable the identification of novel targets and the subsequent development of therapies that are essential for improving quality of life for the millions of individuals suffering with NP. Here we analyzed a publically available microarray dataset (GSE30165) in order to identify new RNAs (e.g., messenger RNA (mRNA) isoforms and non-coding RNAs) underlying NP. GSE30165 profiled gene expression in dorsal root ganglion neurons (DRG) and in sciatic nerve (SN) after resection, a NP model. Gene ontological analysis shows enrichment for sensory and neuronal processes. Protein network analysis demonstrates DRG upregulated genes typical to an injury and NP response. Of the top changing genes, 34 and 36% are associated with more than one protein coding isoform in the DRG and SN, respectively. The majority of genes are receptor and enzymes. We identified 15 long non-coding RNAs (lncRNAs) targeting these genes in LNCipedia.org, an online comprehensive lncRNA database. These RNAs represent new therapeutic targets for preventing NP development and this approach demonstrates the feasibility of data reanalysis for their identification.

## Introduction

The majority of patients with spinal cord injury (SCI) experience chronic pain, with a high percentage experiencing neuropathic pain (NP) (Siddall et al., 1999). NP develops concurrently with anatomical and physiological changes in the peripheral and central nervous system (PNS and CNS). For example, peripheral neuron innervation into the spinal dorsal horn (Nakamura and Myers, 2000) as well as both peripheral and central neurotransmitter expression and excitability change following injury (Chaplan et al., 1997; Fukuoka et al., 1998; Alexander et al., 2012). Identifying gene expression patterns in sensory neurons (i.e., dorsal root ganglion, DRG neurons) under normal and NP conditions is essential to understanding the genetic mechanisms behind the development of NP. Importantly, as the cells involved in NP are still alive, they are viable targets for small molecule or gene therapy approaches aimed at restoring normal function.

RNAs that do not code for a protein, or non-coding RNAs (ncRNAs; e.g., microRNAs: miRNAs and long ncRNAs: lncRNAs), are implicated in many biological and pathological processes such as cancer development, progression, and metastasis (Calin and Croce, 2006; Zhong et al., 2009; Gutschner and Diederichs, 2012; Ziats and Rennert, 2013), and genetic variations within ncRNA loci are increasingly associated with developmental disorders and disease states (Pasmant et al., 2011; Richardson et al., 2011; Zhang et al., 2012). Since RNA-regulated gene expression is increasingly involved in pathological conditions we wanted to understand RNA expression and diversity in the context of NP. Indeed evidence for the involvement of lnc and miRNAs in the development of NP is emerging although in its infancy. For example, *Kcna2 antisense* lncRNA is expressed in DRG neurons and causes or reduces NP through its ability to regulate the voltage-dependent potassium channel, *Kcna2*, impacting neuronal excitability (Zhao et al., 2013). A recent study examined miRNA expression along with gene expression in a sciatic nerve (SN) ligation model of NP (von Schack et al., 2011). The authors found 63 miRNAs changing expression; interestingly the majority (59) of miRNAs were down-regulated in the ipsilateral DRG one level above the injury (von Schack et al., 2011). It is likely that additional ncRNAs contribute to NP development after SCI but identification of these RNAs has remained challenging.

In addition to ncRNAs, messenger RNA (mRNA) isoforms drive distinct biological functions (Hong et al., 2008) and may underlie pathological conditions (Gerstin et al., 1998; Pertin et al., 2005; Dina et al., 2008; Kanzaki et al., 2012). For example, neuregulin-1 has three isoforms that undergo alternative expression regulation (Nrg1 I and II increase and Nrg1 III decreases) after spinal nerve ligation in the rat, changes associated with mechanical sensitivity of the ipsilateral hind paw (Kanzaki et al., 2012). Protein kinase C isoform delta is linked to L-type calcium channel upregulation and may contribute to alcohol-induced peripheral neuropathy (Gerstin et al., 1998; Dina et al., 2008). These findings demonstrate that mRNA isoforms play an important biological role but the paucity of evidence for mRNA isoforms in critical biological roles may in part be due to lack of their complete identification.

Here we sought to identify additional mRNA isoforms and regulatory RNAs contributing to NP development. Multiple methods are available for understanding gene expression (e.g., microarray, RNA-seq) and many laboratories are applying these methods to various pathologies such as SCI and NP. The majority of SCI research is performed in *Rattus norvegicus* (rat) because the injury response and lesion formation are similar to human (Sroga et al., 2003). A search of the Gene Expression Omnibus (GEO) (Edgar et al., 2002; Barrett et al., 2005) database using “drg pain” or “drg NP” as terms produced over 200 results, with the majority of studies in rat using microarrays. We examined several datasets and chose GSE30165 because it examined global gene expression changes after SN resection in both the DRG and SN. We identified the differentially expressed rat genes and then converted them to their mouse homologs using a sequence based strategy, allowing us to identify the associated mRNA isoforms and regulatory RNAs. This strategy globally identifies possible new RNAs for targeting and provides a roadmap for the re-evaluation of already existing datasets.

## Materials and methods

### Sciatic nerve injury

This following procedural guideline was kindly provided by Dr. Bin Yu, Jiangsu Key Laboratory of Neuroregeneration, Nantong University, Nantong, China, the investigator who uploaded the results to the NCBI GEO Database. Briefly, male Sprague-Dawley rats (180–220 g), were anesthetized by an intraperitoneal injection of complex narcotics (85 mg/kg trichloroacetaldehyde monohydrate, 42 mg/kg magnesium sulfate, 17 mg/kg sodium pentobarbital), and the SN was exposed and lifted through an incision on the lateral aspect of the mid-thigh of the left hind limb. A 1 cm long segment of SN was then resected at the site just proximal to the division of tibial and common peroneal nerves, and the incision sites were then closed. To minimize discomfort and possible painful mechanical stimulation, the rats were housed in large cages with sawdust bedding after surgery. L4-6 DRG tissues and SN tissues (0.5 cm) were collected at different time points after injury, respectively. All the experimental procedures involving animals were conducted in accordance with Institutional Animal Care guidelines and ethically approved by the Administration Committee of Experimental Animals, Jiangsu Province, China.

### Gene expression analysis

Gene expression data and analysis was obtained from the NCBI NIH GEO, dataset GSE30165. Sample preparation was described in the dataset design description. Briefly, gene expression levels from L4-6 DRG tissues and proximal SN tissues (0.5 cm) were examined at 0 days, 1 day, 4 days, 7 days, and 14 days after SN resection. This dataset consisted of three samples each for the DRG and SN tissues, and gene expression data was available for all samples at each of the 5 times points. GEO2R was used to compare expression between sham and 1 day post-injury (dpi); sham and 4 dpi; sham and 7 dpi; and finally sham and 14 dpi for both the DRG and SN. GEO2R analyzes gene expression using GEOquery and the Linear Models of Microarray Analysis R package (limma) (Edgar et al., 2002; Gentleman et al., 2004; Smyth, 2004, 2005; Barrett et al., 2005; Davis and Meltzer, 2007). First, GEOquery formats the data into tables for R and then limma R applies the Benjamini and Hochberg False Discovery Rate (FDR) correction for multiple comparisons testing to determine the adjusted *p*-value, *p*-value, moderate *t*-statistic, log fold change, and the moderate F-statistic (Edgar et al., 2002; Barrett et al., 2005; Gentleman et al., 2004; Smyth, 2004; Davis and Meltzer, 2007). We determined the top 250 genes that changed significantly at each time point compared to baseline with an adjusted *p*-value of <0.05 in order to identify the genes that changed over the time-course following injury, and not to identify the most differentially expressed genes across the experiment. We looked at the top 250 differentially expressed genes in each comparison to focus our results to only the genes that changed the most at each time point. The final subset of genes from each comparison was restricted to only those with a fold change in either direction that was greater than 2 for the DRG and SN tissues separately. The final list of genes consisted of all that had at least one time point that showed a change with an adjusted *p* < 0.05 and a fold change of 2, resulting in the identification of 246 genes for the DRG dataset and 549 for the SN dataset. The values at each time point were normalized with respect to the average expression value over all time points for each gene. Heatmaps were generated using the bioinformatics toolbox in Matlab.

### Gene ontology analyses

The final gene list after applying the cutoffs (adj. *p* < 0.05 and fold change of 2) was input into the DAVID Functional Annotation interface and submitted as a gene list selecting species *Rattus norvegicus* (Huang da et al., 2009a,b). Gene Ontology (GO) charts were created using the following options: thresholds: count 2, EASE 0.1; Benjamini correction, Number of records = 1000.

### Rat to mouse conversion

The microarray probe sequences for the differentially expressed genes at different time points following nerve injury were extracted for both DRG and proximal SN tissues from the GEO, Agilent-014879 Whole Rat Genome Microarray 4x44K G4131F. The extracted sequences were then aligned against mouse reference (Ensembl), Mus_musculus.GRCm38.74.cdna.all.fa (Flicek et al., 2013, 2014) using BLAT (Kent, 2002), a fast spliced alignment program. BLAT was executed with blast8 as output and all other parameters set at default values. The alignment was done against mouse reference to identify the homologous sequences between the two rodent species. The aligned rat sequences were then annotated using mouse, Mus_musculus.GRCm38.74.gtf to associate the rat genes from the microarray data against the corresponding mouse homologs based on the alignment results, and then the gene biotypes were assigned based on the mouse annotation provided by Ensembl (Hubbard et al., 2002). Since the rat annotations are not defined as thoroughly as the mouse (Table 1), mouse annotation was chosen to classify the gene biotypes that includes protein-coding and specific type of non-coding.

**Table 1:** **The rat genome has fewer RNA annotations in all categories**.

	**Protein coding**	**Micro**	**Long non-coding**	**Small-nucleolar**	**Small-nuclear**	**Antisense**
*Mus musculus*	22,740	2010	1795	1556	1387	1476
*Rattus norvegicus*	19,878	419	0	0	0	0

*The number of protein coding, micro, long non-coding, small nucleolar, small nuclear, and antisense RNAs found in the Mus_musculus.GRCm38.74.gtf and Rattus_norvegicus.Rnor_5.0.74.gtf from the Ensemble Database*.

### Network analyses

#### Protein interactions (Figure 2)

A very popular tool named STRING (V. 9.1, http://string-db.org/) was used for visualizing interactomes starting from identified differentially expressed entities (genes and transcripts) in both species. In particular the confidence and evidence STRING protein–protein interaction modes were applied.

In confidence view, stronger associations are represented by thicker lines, while in evidence view; different line colors represent the types of evidence for specific associations: expression, binding catalysis, and post-translational modification.

#### Expression interactions (Figure 3)

Mouse gene symbols returned from the rat to mouse conversion were uploaded to Ingenuity® Systems (www.ingenuity.com). Interactions were added using the Connect Tool. Molecules involved in depolarization and nociception were identified using the Overlay Tool. The RNAs with greater than 1 CDS and associated ncRNAs were added by hand.

## Results

### Identification of differentially expressed genes

**Figure 1:**
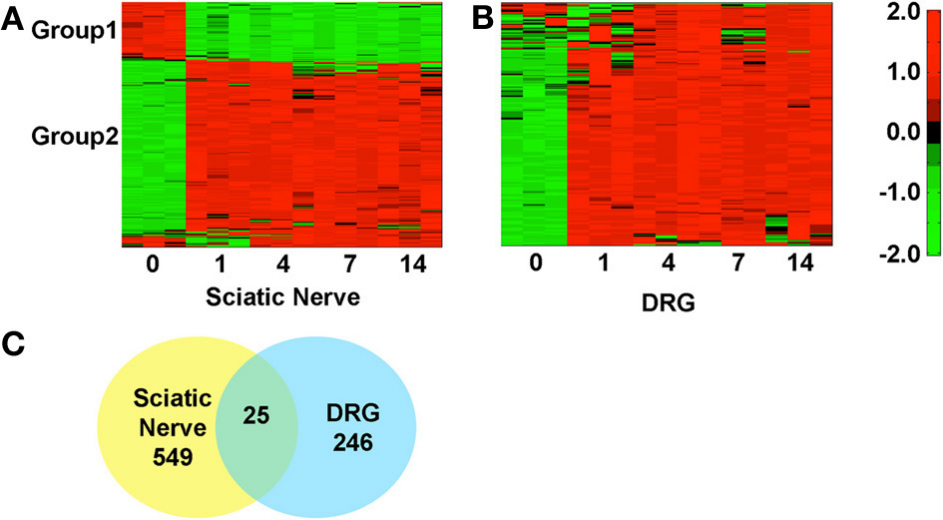
**Hierarchical clustering of normalized expression values at various days post-sciatic nerve resection in the sciatic nerve and in the DRG**. The heatmaps reflect gene expression values normalized to the mean across all time points (day 0, 1, 4, 7, and 14 post-injury) for genes that met the cutoff in at least one time point (*p* < 0.05 and fold change >2). **(A)** In the SN there are two distinct groups, 1 and 2, which decrease (green) or increase (red) in expression, Supplementary Table 2. **(B)** In the DRG the majority of genes decrease in expression. **(C)** Some genes overlap (25) between SN and DRG but the majorities of changing genes were unique to each tissue.

We identified the top 250 IDs from the microarray dataset that met our cutoffs for a significant expression change (adjusted *p* < 0.05 and fold change >2; Figures 1A,B). There were 549 unique IDs corresponding to 366 rat genes with gene symbols in the SN and 246 unique IDs corresponding to 158 rat genes with gene symbols in the DRG (Figure 1C; Supplementary Tables 1, 2). 25 of the top changing were found in both samples, 18 of which had associated gene symbols (Supplementary Table 3). In the SN, a subset of genes decreased expression (Group 1, Figure 1A; Supplementary Table 1), while the bulk increased in expression (Group 2, Figure 1A; Supplementary Table 1). In the DRG, the majority of genes increased in expression (Group2, Figure 1B; Supplementary Table 2). These data indicate major gene expression changes in the SN and in the DRG after injury.

### Gene ontology analysis

A GO term enrichment analysis (Huang da et al., 2009a,b) was subsequently performed to gain a deeper understanding of these genes. GO enrichment analysis assigns general descriptions based on biological function, cellular component, and molecular function, to groups of genes. We isolated the up or down regulated genes (SN: Group 1–3, Supplementary Table 1; DRG: Group 1 and 2, Supplementary Table 2) and performed GO analysis using DAVID Bioinformatics Resource v6.7 (Huang da et al., 2009a,b). GO analysis on the down-regulated genes in the SN sample show the majority of biological processes are biosynthetic and catabolic functions while the majority of the up-regulated processes are related to the detection of stimuli and signaling responses (Supplementary Table 4). Not surprisingly, the majority of cellular components up- or down-regulated are associated with the cytoplasm and cellular membrane (Supplementary Table 4). The majority of molecular functions switch from ion binding (downregulated) to chemokine and enzymatic activities (upregulated; Supplementary Table 5). These data suggest a switch from neurotransmission and normal sensory functioning to immune response detection and receptor activation, consistent with a switch from normal sensory neurotransmission to an injury response in the SN. In the DRG sample, the majority of genes were upregulated after injury (Figure 1B). Most biological processes in the DRG upregulated genes fall into signaling pathways (e.g., G-protein, neuropeptide) or detection and reaction to stimuli (e.g., sensory perception of chemical stimulus, inflammatory response; Supplementary Table 5). In cellular component, the majority associated with the membrane, extracellular space, and nerve terminal (cellular component, Supplementary Table 6) and the molecular functions are associated with receptors, cytokines, or hormone activity (molecular function, Supplementary Table 5). These data suggest a major change in DRG gene expression in areas directly associated with NP development such as neurotransmission and receptor expression (Xu et al., 1993, 2007; Fukuoka et al., 1998; Sah et al., 2003; Pertin et al., 2005; Mika et al., 2008; Miller et al., 2009).

### Identification of isoforms

During the analysis it was observed that many rat UniqueIDs were not associated with a gene name or symbol (Supplementary Tables 2, 3). Indeed the rat genome contains far fewer elements compared to the mouse (Table 1). This suggests that using the rat for gene array and/or RNA-seq experiments is problematic and could severely limit gene expression analysis interpretation. To address this problem and gain insight into gene expression and regulation we converted the rat genes (Figure 1) to their mouse homologs using a sequence based strategy (Methods; Supplementary Tables 6, 7). BLAT finds similar sequences of length 25 base pairs or greater. We set a homology threshold of 84% and higher to extract the potential homologs from the BLAT output using the default parameters. We retrieved the corresponding target mouse gene names from the BLAT output and used them for downstream analysis. Using this homology-based strategy we identified 455 corresponding mouse genes in SN and 167 in the DRG (Supplementary Tables 6, 7). These genes give rise to hundreds of isoforms and produce multiple protein isoforms (Table 2). Isoform switching [aka: alternative open reading frame (ORF) utilization], is one mechanism driving neural development (Ruusuvuori et al., 2004; Bani-Yaghoub et al., 2007) and contributing to disease states in the body (Periasamy and Kalyanasundaram, 2007). It could be a potential mechanism underlying NP development. We identified numerous differentially expressed genes whose isoforms differ at the level of the coding DNA sequence (CDS) leading to alternative ORFs (Table 2). Protein coding differences were most abundant in enzymes, ion-channels, transcription regulators, and G-protein coupled receptors (Table 3), all highly associated and implicated in NP.

**Table 2:** **Differentially expressed genes have abundant transcript diversity**.

	**SN**	**DRG**
Genes	445	167
Transcripts	1451	409
Transcripts with different CDS	162	36

*Mouse transcript information was obtained from the Ensemble Mus_musculus.GRCm38.74.gtf. The number of genes, transcripts and transcript harboring changes in the coding DNA sequence (CDS) was identified*.

**Table 3:** **Enzymes and transcription regulators are associated with the most protein coding isoforms in the SN and DRG, respectively**.

	**SN**	**DRG**
Enzyme	49	3
G-protein coupled receptor	6	5
Ion channel	12	1
Kinase	5	1
Peptidase	7	2
Transcription regulator	2	8
Translation regulator	1	1
Transmembrane regulator	5	2
Transporter	1	1

*Categories were assigned using the molecular annotations feature in Ingenuity® Systems, www.ingenuity.com*.

### Network analysis and ncRNA regulation prediction

**Figure 2:**
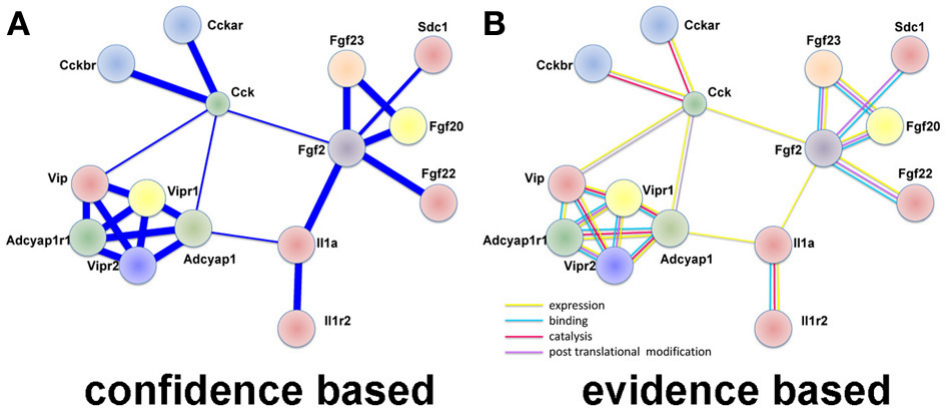
**Protein interaction network using LINC identifies neuropeptide interactions after injury in the DRG**. **(A)** A confidence view of protein interactions. Thicker lines represent stronger associations. **(B)** An evidence view of protein interactions. Both observations support previous studies demonstrating increased expression of neuropeptides after injury. In particular, it is noticed the modular connectivity centered on VIP, ADCYAP1, and CCK, all appearing up-regulated in DRG after injury and associated with NP. Evidence is taken from reports in the literature. Yellow: expression; blue: binding; lilac: violet: catalysis; and post-translation modification. This observation supports previous studies demonstrating increased expression of neuropeptides after injury.

In large datasets relationships between differentially expressed genes are uncovered by examining protein-protein interactions. We used STRING (Franceschini et al., 2013), which utilizes both known and predicted protein associations to generate protein interaction networks. In DRG up-regulated genes, several direct protein interactions among molecules known to change expression after DRG neuron injury were uncovered. The most prominent group of interactions in this analysis was between the neuropeptides vasoactive intestinal peptide (VIP), its receptors (VIPR1/2), pituitary adenylate cyclase-activating polypeptide (ADCYAP1 aka PACAP), its receptor (ADCYAP1R1), and cholecystokinin (CCK) and its receptors (CCKAR, CCKBR; Figure 2). VIP, ADCYAP1, and CCK are upregulated in DRG after injury and are associated with NP (Nielsch and Keen, 1989; Xu et al., 1993; Ma and Bisby, 1998; Ohsawa et al., 2002). These observations support the involvement of these neuropeptides in NP development and support that this dataset is reflecting gene expression changes regulating NP. Interestingly, these neuropeptide receptors have multiple isoforms (Bokaei et al., 2006; Nachtergael et al., 2006), but to date no studies have examined their function in NP models.

**Figure 3:**
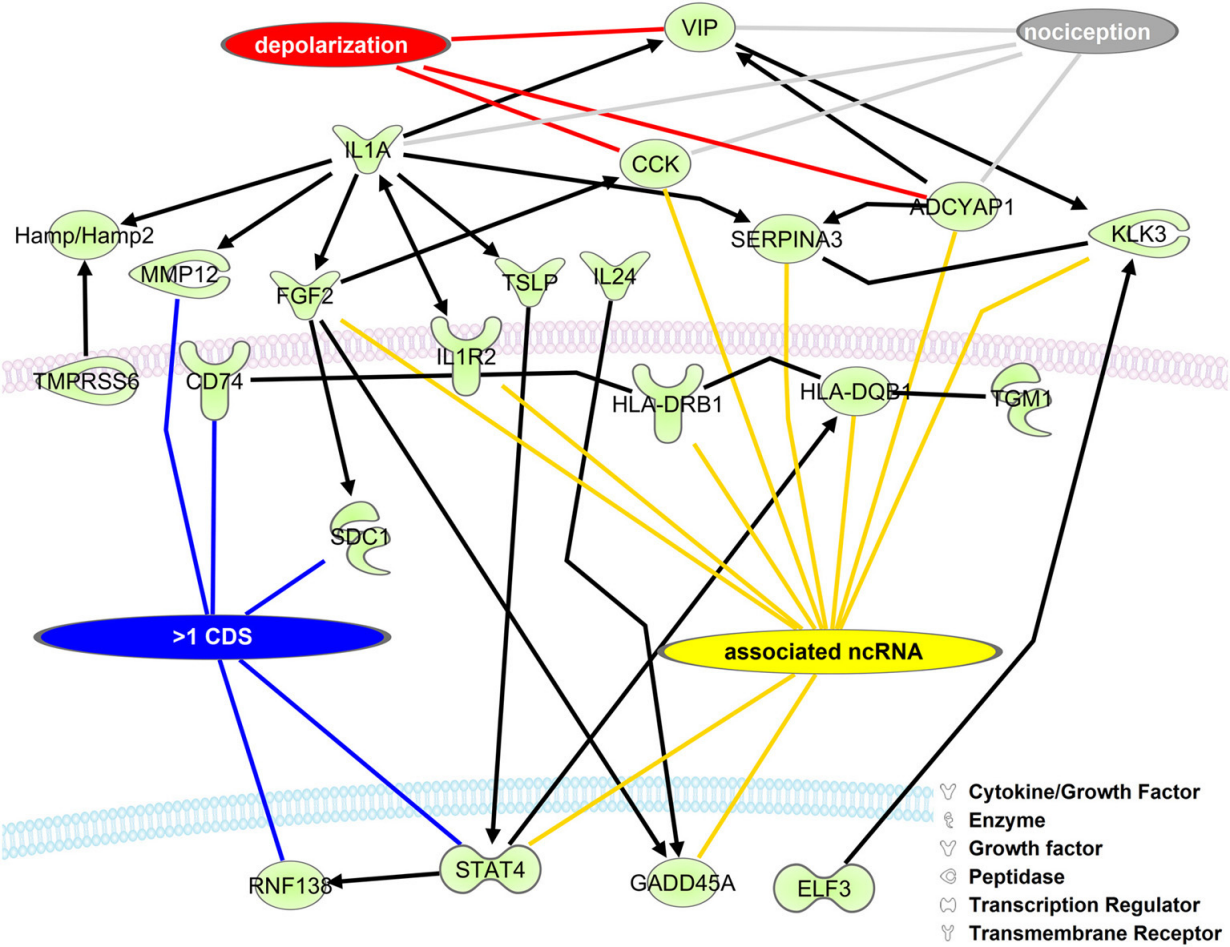
**Upregulated DRG neuronal network is associated with mRNA isoforms and ncRNAs**. The list of mouse homolog DRG upregulated genes (Group 2, Supplementary Table 2) was put into a direct interaction network (Ingenuity® Systems, www.ingenuity.com). Genes having more than one CDS (blue line), an associated ncRNA (yellow line), involved in depolarization (red line), and/or nociception (gray line) are indicated. Black lines with arrows indicate expression activation. Straight black lines indicate protein-protein interaction. ADCYAP1, adenylate cyclase activating polypeptide 1; CCK, cholecystokinin; CD74, CD74 molecule, major histocompatibility complex, class II invariant chain; ELF3, E74-like factor 3; FGF2, fibroblast growth factor 2; GADD45A, growth arrest and DNA-damage-inducible, alpha; Hamp/Hamp2, hepcidin antimicrobial peptide; HLA-DQB1, major histocompatibility complex, class II, DQ beta 1; HLA-DRB1, major histocompatibility complex, class II, DR beta 1; IL1A, interleukin 1, alpha; IL1R2, interleukin 1 receptor, type II; IL24, interleukin 24; KLK3, kallikrein-related peptidase 3; MMP12, matrix metallopeptidase 12; RNF138, ring finger protein 138; E3 ubiquitin protein ligase,: syndecan 1; SERPINA3, serpin peptidase inhibitor, clade A, member 3; STAT4, signal transducer and activator of transcription 4; TGM1, transglutaminase 1; TMPRSS6, transmembrane protease, serine 6; TSLP, thymic stromal lymphopoietin; VIP, vasoactive intestinal peptide.

The role of RNA isoforms and their contributions to neuronal development and pathology is slowly being elucidated (Gerstin et al., 1998; Pertin et al., 2005; Dina et al., 2008; Hong et al., 2008; Kanzaki et al., 2012; Lerch et al., 2012b) but a full understanding of RNA isoform diversity is broadly lacking. To identify mRNAs with alternative CDS's with the potential to impact NP development we created a network of DRG enriched genes with the ability to directly regulate each other's expression (Figure 3). As expected, many genes have a role in neuronal depolarization (Jarvis et al., 1995; Beaudet et al., 2000) and nociception (Jeftinija et al., 1982; Mika et al., 2008; Belcheva et al., 2009), two properties of sensory neurons altered in NP states (Chaplan et al., 1997; Fukuoka et al., 1998; Alexander et al., 2012). We highlight genes with more than one CDS because alternative CDS's leads to changes in functional protein domains which alter cellular function.

LncRNAs have recently been demonstrated to regulate sensory neuronal excitability and NP (Zhao et al., 2013). To identify potential additional gene targets for regulation we searched a database of lncRNAs (Volders et al., 2013). The nomenclature for lncRNAs in this database makes searching straightforward. Transcripts overlapping one or more exons are named with the same gene symbol and therefore considered the same gene (Volders et al., 2013). Searching gene symbols identifies associated lncRNAs. We found 15 lncRNAs conserved between human and mouse in our dataset that corresponded to significantly changing genes (Supplementary Table 8). There were an additional 11 lncRNAs not identified as conserved across species (http://www.lncipedia.org/db/search). Given that lncRNAs have a high degree of evolutionary conservation (Qu and Adelson, 2012); it is possible these additional genes are regulated similarly in rats and mice (Figure 3). The genes identified with a potential lncRNAs fall into many categories such as enzymes (HSD3B2 and PDE6B), growth factors (FGF2), transmembrane receptors (CHRNA1, HLA-DRA, HLA-DRB1, IL1R2, and SEMA6A), and transcriptional regulators (NKZ6.2, SOX11, and STAT4). This demonstrates that lncRNA regulation of gene expression is likely not limited to one particular gene category or class of protein. These strategies highlight a way to reanalyze existing data and extend it to identify novel mRNA isoforms and regulatory RNAs to further our understanding of NP and can be extended to other disease datasets.

## Discussion

Millions of people worldwide, including the majority of SCI patients, experience NP. The prevalence of NP and the minimal availability of effective treatment options make the identification of the molecular pathways leading to NP development a high priority. The majority of studies examining gene expression changes in NP models use a microarray approach (except one study, GSE53768, released 01/07/2014 which used RNA-seq and was unpublished at the time paper submission). Therefore, the identification of all expressed RNAs (e.g., isoforms and regulatory RNAs) is lacking, omitting numerous potential therapeutic targets. To identify RNA isoforms and regulatory RNAs relevant to NP we examined differentially expressed genes from a publically available microarray study using a rat NP model (GSE30165). We identified over 200 genes significantly changing in DRG neurons and over 400 in the SN (Figure 1; Supplementary Tables 1–3). Differentially expressed genes in this dataset show GO enrichment for inflammatory processes, critical regulators and contributors to NP (Supplementary Tables 4, 5; Hulsebosch, 2008; Kigerl et al., 2009; Alexander et al., 2012).

One challenge of this dataset was the use of a rat model system. We suggest that genetic studies should be performed in mice given that the rat genome annotation is vastly incomplete (Table 1). Given the lack of annotation, our ability to identify mRNA isoforms and ncRNAs from the rat database was limited. Therefore, we retrieved differentially expressed rat RNA sequences (Supplementary Tables 1, 2), and took mouse RNAs with an 84% and greater homology to the rat sequences and then examined these sequences for RNA isoforms and potential regulatory RNAs. We identified 455 mouse genes in SN, 167 in DRG, and thousands of RNA isoforms for each gene (Supplementary Tables 6, 7). We created a network of the interacting up-regulated genes from the DRG dataset. Interestingly, in this dataset we identified 15 conserved lncRNAs that could regulate these transcripts in the rat or mouse (Figure 3, Supplementary Table 8). LncRNAs regulate protein coding gene expression by affecting DNA organization (e.g., defining chromatin domains; Rinn et al., 2007), transcription (Zhao et al., 2013), and/or post-transcription processing (Mercer et al., 2009). Most lncRNAs are associated with a decrease in their target's expression [e.g., HOTAIR's repression of the HoxD locus (Rinn et al., 2007); Kcna3 antisense repression of Kcna3 (Zhao et al., 2013)]. There is a single compelling example of an lncRNA regulating NP development. Kcna3 antisense expression increased after peripheral nerve injury, increased neuronal excitability, and when overexpressed induced NP pain symptoms (Zhao et al., 2013), a remarkable effect for a single lncRNA. One area of future investigation is to determine global lncRNA expression changes after SCI, because while Kcnc3 antisense expression increased, it is just as likely that some lncRNAs expression would decrease. In this study we found that SNI in the DRG led to a majority of genes increasing expression (Figure 1). Therefore, it is possible that SNI causes a reduction in the lncRNAs we identified (Figure 3) that contributed to their target gene expression increase (Figure 1). In addition, we hypothesize that these lncRNAs represent therapeutic targets since overexpressing them would repress their target genes and potentially reduce NP symptoms. For example, the increases in interleukin 1 receptor (IL1R), adenylate cyclase activating polypeptide 1 (ADCYAP1), and cholecystokinin (CCK) may be associated with a decrease in their associated lncRNAs (Figure 3). This interaction, if occurring, may contribute to their roles in nociception (Figure 3; IL1R through binding to IL1A and ADCYAP1 through VIP binding; Jeftinija et al., 1982; Xu et al., 1993; Mika et al., 2008). We acknowledge that while these are intriguing possibilities, all of these isoforms and lncRNAs require functional studies to test if they are viable candidates, but note that identification is the first step toward determining functional relevance.

NP is debilitating and in need of better therapeutic strategies. A multitude of well-controlled publically available data exists in the GEO database. We identified isoform diversity and potential ncRNAs through a data reanalysis using a straightforward bioinformatic approach. There is growing evidence that RNA isoforms and lncRNAs are important regulators of cellular function and contribute to pathological processes (Gerstin et al., 1998; Hong et al., 2008; Kanzaki et al., 2012; Lerch et al., 2012b). Future studies will employ RNA-seq enabling full scale detection of all RNAs within a cell type (Faghihi and Wahlestedt, 2009; Lerch et al., 2012a,b) giving a complete picture of gene expression but here we demonstrate a fast and economical way to find new targets underlying NP development.

### Conflict of interest statement

The authors declare that the research was conducted in the absence of any commercial or financial relationships that could be construed as a potential conflict of interest.
